# *Eriocaulon buergerianum *extract protects PC12 cells and neurons in zebrafish against 6-hydroxydopamine-induced damage

**DOI:** 10.1186/1749-8546-6-16

**Published:** 2011-04-28

**Authors:** Meiwei Wang, Zaijun Zhang, Lorita Chi-Veng Cheang, Zhixiu Lin, Simon Ming-Yuen Lee

**Affiliations:** 1 State Key Laboratory of Quality Research in Chinese Medicine, Institute of Chinese Medical Sciences, University of Macau, Av. Padre Tomás Pereira, Taipa, Macao, China; 2 School of Chinese Medicine, The Chinese University of Hong Kong, Shatin, NT, Hong Kong, China

## Abstract

**Background:**

*Ericaulon buergerianum *(*Gujingcao*) is an ophthalmic, anti-inflammatory and antimicrobial Chinese medicinal herb. This study aims to investigate the neuroprotective effects of *Ericaulon buergerianum *ethanol extract (EBE) and to elucidate its underlying action mechanism.

**Methods:**

The viability of dopaminergic (DA) neuron in zebrafish was examined by anti-tyrosine hydroxylase (TH) immunostaining. The locomotor activity of zebrafish was assessed with a digital video tracking system. The viability and cellular damage of the PC12 cells were determined by MTT and LDH assays respectively. The nuclear morphological changes in apoptotic cells were evaluated with DNA staining by Hoechst 33342 dye. Intracellular nitric oxide (NO) was quantified by DAF-FM diacetate staining. The expression of inducible nitric oxide synthase (iNOS) was determined by Western blot.

**Results:**

EBE inhibited the 6-OHDA-induced decrease in total distance of movement in zebrafish. Pretreatments of EBE (25, 50, 100 and 200 μg/ml) increased the viability of 6-OHDA-damaged PC12 cells in a dose dependent manner. Protection against 6-OHDA-induced nuclear fragmentation and accumulation of apoptotic bodies was also observed in EBE pretreated cells. Anti-oxidative (inhibition of NO production and iNOS expression in PC12 cells *in vitro*) activities of EBE are related to its neuroprotective effects in 6-OHDA-induced DA neuron damage.

**Conclusion:**

EBE exhibited significant neuroprotective activities in zebrafish, including recovery of dopaminergic neuron loss caused by 6-OHDA in a dose-dependent manner *in vivo*, inhibition of 6-OHDA-induced decrease of total distance in movement in zebrafish. The iNOS-NO pathway may be involved.

## Background

A hydroxylated analogue of dopamine, namely 6-Hydroxydopamine (6-OHDA) which induces damage of dopaminergic neurons *in vivo *and *in vitro*, is commonly used in model systems to mimic Parkinson's disease of which the main neuropathological feature is the loss of *substantia nigra pars compacta *(SNpc) dopaminergic (DA) neurons. The toxic effects of 6-OHDA are mainly attributed to the formation of free radicals, inflammatory processes and apoptosis [1,2].

Early studies indicated that NO participated in cellular signaling pathways regulating broad aspects of brain functions, such as synaptic plasticity, normal development and neuronal cell death [3]. NO is synthesized from L-arginine by nitric oxide synthase (NOS). Among the three major isoforms of NOS, inducible NOS (iNOS), a calcium-independent isoform, is regulated by oxidative stress and some inflammatory cytokines [4]. The implication of NO in PD pathogenesis is supported by the observations of unregulated iNOS expression in activated microglia [5,6]. NO induces neuronal cell damage by disrupting neuronal mitochondrial electron transport chain function [7,8]. As a result, the agents recovering the impaired mitochondrial function, suppressing neuroinflammation and the production of NO and iNOS may be beneficial for PD patients.

*Ericaulon buergerianum *(*Gujingcao*), an aquatic plant native to Mainland China (*eg *Zhejiang, Guangdong and Fujian provinces), Taiwan and Japan, is used in Chinese medicine as an anti-inflammatory and antimicrobial agent [9]. According to Chinese medicine theories, it expels *Feng *(Wind), clears *Re *(Heat) and brightens the eyes. In Chinese Pharmacopoeia (2005), the capitulum of *Ericaulon buergerianum *is one of the most frequently used Chinese medicinal herbs, with flavonoids, volatile oils, anthraquinone, naphthopyranones, protocatechuic acid and c-tocopheryl acetate being the bioactive constituents [10]. Flavonoids such as patuletin hispidulin, quercetin, quercetagetin and quercetagetin derivatives and volatile oil such as palmitic acid, (*Z*, *Z*)-9, 12-octacosane-dienoic acid are the two major classes of chemicals in *Ericaulon buergerianum *[9,11]. Water extract of *Ericaulon buergerianum *exhibits antimicrobial properties [11]. *Ericaulon buergerianum *demonstrates significant therapeutic effects on headache, toothache, nasosinusitis, night blindness, glaucoma, retinochoroiditis, conjunctivitis and other eye diseases [12]. *Guqing Tang*, a Chinese herbal formula consisting of *Ericaulon buergerianum *and *Celosia argentea *(*Qingxiangzi*), is used to treat headache and eye diseases [13].

The value of zebrafish (*Danio rerio*) for drug screening, target validation and toxicological studies is increasingly recognized in recent years [14,15]. A region in the zebrafish brain anatomically corresponding to the striatum was identified in the forebrain [16]. Zebrafish also display learning, sleeping, drug addiction and neurobehavioral phenotypes that are quantifiable and related to those in humans [17,18]. Therefore, it is an ideal model to study the neuroprotective effect of herbal medicine *in vivo*.

The present study aims to investigate the neuroprotective effects of *Ericaulon buergerianum *in PC12 cells and zebrafish and elucidate the underlying mechanism of the protective effects.

## Methods

### Chemicals and reagents

Heat-inactivated horse serum, fetal bovine serum (FBS), penicillin and streptomycin were purchased from Gibco Invitrogen (USA). Nutrient Mixture F12 Ham Kaighn's Modification (F-12 K) growth medium, dimethyl sulfoxide (DMSO) and 3-(4,5-dimethylthiazol-2-yl)-2,5-diphenyltetrazolium bromide (MTT) were purchased from Sigma (USA). Cytotoxicity Detection Kit was purchased from Roche Applied Science (Germany). The fluorescent probe 4-amino-5-methylamino- 2',7'-difluorofluorescein diacetate (DAF-FM diacetate) and Hoechst 33342 dye were purchased from Molecular Probes(USA). RIPA lysis buffer, PMSF, protease inhibitor cocktail and BCA protein assay kit were purchased from Pierce Biotechnology (USA). Polyvinylidene difluoride (PVDF) membrane was purchased from Bio-Rad (USA). Anti-iNOS was obtained from Cell Signaling Technology (USA). ECL advanced western blotting detection kit was purchased from Amersham (UK). All other reagents used in this study were obtained from Sigma.

### Extraction procedure

Crude herb of *Ericaulon buergerianum *was purchased from ZhiXin Chinese Pharmaceutical Company in Hong Kong and authenticated in the School of Chinese Medicine, The Chinese University of Hong Kong according to appearance identification of raw material and comparison of chemical constituents which have described in Zhong-Yao-Zhi [19]. The crude herb was first ground into powder with an electric grinder (Yongkang Weifeng Electric Co. Ltd., China). The powder (100 g) was then extracted with 80% ethanol for two hours, and the extract was filtered and dried. *Ericaulon buergerianum *ethanol (EBE) extract (10.8 g) was stored at -20°C.

### Chemical analysis of Ericaulon buergerianum ethanol extract

EBE was dissolved in DMSO (100 mg/ml) and filtered with a 0.45 μm membrane filter. Then the filtrate was analyzed on an Agilent 1100 series chromatographic system (Agilent, USA) consisting of a vacuum degasser, a binary pump, an auto-sampler, a column oven and a diode array detector (DAD). Chromatographic separation was achieved on a GL Sciences Inertsil ODS-4 column, 5 μm, 250 mm × 4.6 mm i.d. (GL Sciences, USA) at ambient temperature. The flow rate was 0.8 mL/min. The mobile phase consisted of Milli-Q water (A) and acetontrile (B). The gradient elution was as follows: 10-35% (B) in 0-40 min, 35-50% (B) in 40-50 min and 50-100% (B) in 50-60 min. Detection wavelength was set at 280 nm. Injection volume was 10 μL. Evaluation of UV data was performed on an Agilent ChemStation A.09.03 (Agilent, USA) and DataAnalysis 2.2 (Bruker Daltonics, USA).

### Fish maintenance

Embryos of wild-type (AB strain) zebrafish were collected after natural spawning, staged according to standard criteria [20], and synchronously raised at 28.5°C. Embryos were maintained in embryo medium (13.7 mM NaCl, 540 μM KCl, pH7.4, 25 μM Na_2_HPO_4_, 44 μM KH_2_PO_4_, 300 μM CaCl_2_, 100 μM MgSO_4_, 420 μM NaHCO_3_, pH7.4). Since embryos received nourishment from the attached yolk sac, no additional maintenance was required.

### Cell culture

Stock cultures of rat pheochromocytoma cells (PC12) (CRL-1721) were purchased from American Type Culture Collection (ATCC, USA). They were cultured in F-12 K supplemented medium with 15% (v/v) heat-inactivated horse serum, 2.5% (v/v) FBS, penicillin (100 U/ml) and streptomycin (100 μg/ml) in a humidified atmosphere of 5% CO_2 _at 37°C. The medium was changed every other day.

### Experimental design

Fertilized eggs obtained from mating pairs of adult zebrafish were cultured in embryo medium. All experiments were performed in 12-well plates with 20 embryos in each well. Phenylthiourea (PTU, Sigma-Aldrich, USA) was added for a final concentration of 0.003% to prevent pigmentation of embryos, up to 2 day post fertilization (dpf) and larvae (>2 dpf). 2 dpf zebrafish was exposed to 250 μM 6-OHDA and different concentrations (25, 50, 100 μg/ml) of EBE for 24 hours. Zebrafish co-treated with 6-OHDA and Nomifensin (DAT inhibitor)/L-nitroarginine methylester (L-NAME) were used as positive controls. Anti-TH immunostaining was performed to evaluate the viability of dopaminergic neuron in the brain of zebrafish.

PC12 cells were plated at a density of 10^4 ^cells/100 μl/well in 96-well plates. EBE of different concentrations (25, 50, 100 and 200 μg/ml) was added as pretreatment to PC12 cells incubated in F-12 K medium supplemented with 0.5% (v/v) heat-inactivated horse serum for 12 hours at 37°C. Cells pretreated with L-NAME (250 μM) for 12 hours served as positive controls. The mediums were then discarded, and the cells were incubated for another 12 hours with 6-OHDA (1 mM) dissolved in 0.5% (v/v) heat-inactivated horse serum at 37°C. A control group of untreated normal cells was also included. The viability and cellular damage of the PC12 cells were detected by the MTT and LDH assays respectively.

### Anti-tyrosine hydroxylase (TH) whole mount immunostaining

Zebrafish were fixed in 4% paraformaldehyde in phosphate buffered saline (PBS) for five hours, rinsed and stored at -20°C in 100% EtOH. Fixed samples were blocked (2% lamb serum in phosphate buffered saline containing 0.1% Tween-20 (PBS-T), 0.1% BSA) for one hour at room temperature. A mouse monoclonal anti-tyrosine hydroxylase antibody (1:200 diluted in blocking buffer, MAB318, Millipore, USA) was used as the primary antibody and incubated with the sample overnight at 4°C. Samples were then washed with PBS-T six times (30 min for each wash), followed by incubation with secondary antibody according to the instruction of Vectastain ABC kit (Vector Laboratories, USA). After being stained, zebrafish were flat mounted with 3.5% methylcellulose and photographed.

### Testing of the locomotor activity

Fish behavior was analyzed with a digital video tracking system (Viewpoint, ZebraLab, France). The system consists of a digital video camera connected to a computer system running the analysis software ZebraLab Man Rev 3.6B (ZebraLab, France). The locomotor activity of zebrafish larvae was assessed in a 96 well plate filled with 200 μl embryo medium. At 7 dpf, the larvae were allowed to habituate to the new environment for one hour and their behavior was recorded for two minutes with the Viewpoint ZebraLab system. Total distance of movement of each fish per group was calculated.

### MTT assay

3-(4, 5-dimethyl-2- thiazolyl) 2, 5-diphenyl-2H-tetrazolium bromide (MTT) is a tetrazolium salt that can be reduced to purple-colored formazan by live cells. Formazan is dissolved and the resulting solution can be spectrophotometrically measured. Cells were incubated for four hours at 37°C with MTT solution (0.5 mg/ml) prepared in fresh 0.5% (v/v) heat-inactivated horse serum. The medium was then discarded, and 100 μl of DMSO was applied to each well to dissolve the violet formazan crystals in intact cells. The absorbance was measured at the wavelength of 490 nm by a multi-label counter (Wallac VICTOR3™V, Perkin Elmer, Netherlands). Cell viability was expressed as a percentage of the control (untreated cells). All assays were performed in eight replicates and repeated at least three times.

### LDH assay

Cell viability was determined by the activity of lactate dehydrogenase (LDH) released into the incubation medium when cellular membranes are damaged. Cells were seeded in 96-well plates. After treatments, the released LDH was measured according to the specifications of Cytotoxicity Detection Kit (Roche, Germany). Briefly, 100 μl of culture medium was collected from each well. The absorbance of the medium was measured at 490 nm with 690 nm as a reference wavelength in an automatic microplate reader (Wallac VICTOR3TMV, perkinelmer, USA). Results are shown as percentage versus 6-OHDA group.

### Hoechst 33342 staining

The nuclear morphological changes in apoptotic cells were evaluated by DNA staining with Hoechst 33342 dye. Under fluorescent microscope, Hoechst 33342 stains the condensed chromatin in apoptotic cells much more brightly than in normal cells. Cells were plated into a 12-well plate at 10^5^/well. Cells were pretreated with EBE (50, 100, 200 μg/ml) for 12 hours, then treated with 6-OHDA (1 mM) for 8 hours. Cells were then stained with Hoechst 33342 (10 μg/ml) with RNase (5 μg/ml) in PBS for 15 min at room temperature, followed by a 15-min fixation in 1% (w/v) paraformaldehyde. Images were recorded with a fluorescent microscope (Carl Zeiss, Axiovert 200, USA) with a mounted camera (Carl Zeiss, AxioCam HRc, USA).

### Intracellular NO staining

Intracellular NO was evaluated by the fluorescent probe 4-amino-5-methylamino- 2',7'-difluorofluorescein diacetate (DAF-FM diacetate). DAF-FM diacetate is cell-permeant and passively diffuses across cellular membranes. Once inside cells, it is deacetylated by intracellular esterases to become DAF-FM. The cells were seeded in 96-well black-bottom clear plates. After pretreated with EBE and 6-OHDA for 12 hours and one hour respectively, the cells were washed in PBS and incubated in medium with PBS plus 2.5 μM DAF-FM diacetate for 30 min at 37°C in darkness. Then the cells were washed twice with PBS and the fluorescence was evaluated in a microplate reader (Wallac VICTOR3TMV, perkinelmer, USA) at 495 nm (excitation) and 515 nm (emission). Meanwhile, images were recorded by a fluorescent microscope (Carl Zeiss, Axiovert 200, USA) with a mounted camera (Carl Zeiss, AxioCam HRc, USA).

### Western blot analysis

After treatment, PC12 cells were washed three times with cold PBS and then incubated on ice with RIPA lysis buffer with 1% PMSF and 1% protease inhibitor cocktail for 30 min. Cell lysates were centrifuged at 12,500 × *g *(Hitachi, Japan) for 20 min at 4°C. The supernatant was separated and the protein amount was determined by the BCA protein assay kit. Sample buffer (10% SDS, 250 mM Tris-HCL 6.8, 50% glycerol, 8% DTT, 0.002% blue-bromophenol) was added and mixed with protein at a ratio of 1:4, and boiled at 95°C for five minutes. Protein samples (40 μg) were separated by 10% SDS-poly-acrylamide gel electrophoresis and then transferred to a polyvinylidene difluoride (PVDF) membrane (Bio-Rad, USA) for 90 min at 20 V. Subsequently, the membrane was blocked with 5% non-fat milk in PBS containing 0.1% Tween20 (PBST) for one hour at room temperature. The blots were incubated overnight at 4°C with various primary antibodies (Cell Signaling, USA) including anti-iNOS (1:1000). After three washes with PBS-T, the membranes were incubated with horseradish peroxidase-conjugated secondary antibodies (1:2000) in PBS-T with 5% non-fat milk for one hour at room temperature. After repeated washes, proteins were visualized with an ECL advanced Western blotting detection kit (Amersham, UK) according to the manufacturer's protocol. Protein bands were photographed by a Molecular Imager ChemiDoc XRS (Bio-Rad, USA).

### Statistical analysis

Data are represented as mean ± standard deviation (SD). One-way ANOVA was used to detect significant differences among concentration groups in the experiments. Newman-Keuls Multiple Comparison Test was used to test the statistical significance of the difference between the concentration group and the control (vehicle) group. GraphPad Prism statistical software (GraphPad Software, USA) was used for all calculations. *P *< 0.05 was considered as statistically significant.

## Results and Discussion

### Quality control of EBE

**Figure 1:**
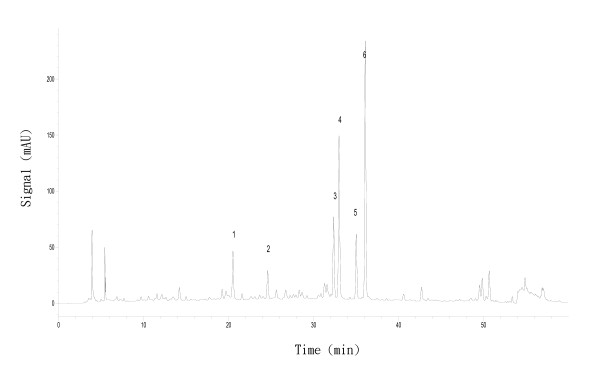
**HPLC/UV chromatogram of *Ericaulon buergerianum *ethanol extract**. Column: ODS-4 column; The flow rate: 0.8 mL/min; The mobile phase consisted of Milli-Q water (A) and acetontrile (B) with a gradient elution of 10-35% (B) in 0-40 min, 35-50% (B) in 40-50 min and 50-100% (B) in 50-60 min.

High-performance liquid chromatography (HPLC) coupled with (DAD) was used to generate the chemical profile of EBE (Figure 1). The HPLC fingerprinting may be used as a reference for the purpose of quality assurance for any future experiments related to EBE.

### Protection of 6-OHDA-induced dopaminergic neuron loss in zebrafish

**Figure 2:**
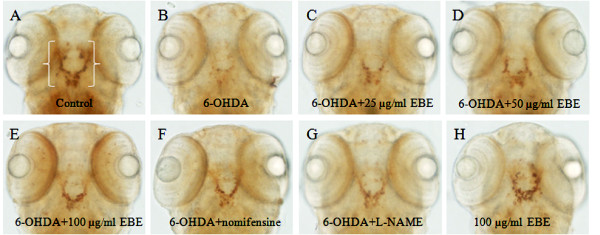
**EBE recovered 6-OHDA-induced dopaminergic neuron loss in zebrafish**. (A-H) 2 dpf zebrafish was exposed to 250 μM 6-OHDA and different concentrations of EBE or 100 μM Nomifensin or 100 μM L-NAME or 100 μg/mL EBE for 24 hours, except the control. The viability of dopaminergic neurons of the zebrafish (indicated by white brackets) was evaluated with anti-tyrosine hydroxylase (TH) immunostaining. Ventral view: anterior to the top.

**Figure 3:**
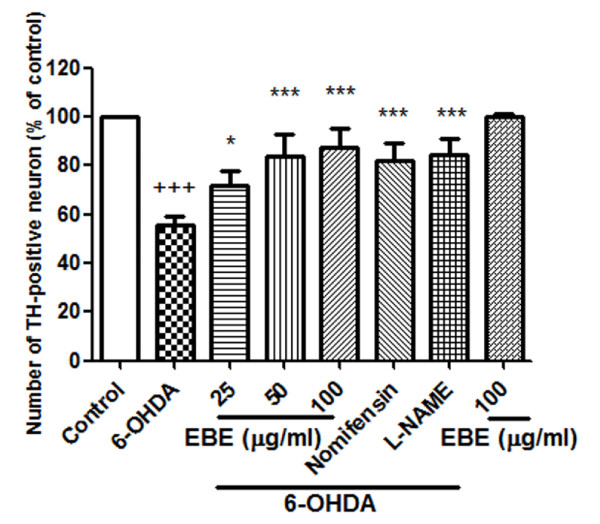
**Quantitative analysis of area of TH^+ ^neuron in zebrafish brain**. All data expressed as percentage of control group, each bar represents mean ± SD. ^+++ ^*P *< 0.0001 versus control group (without 6-OHDA treatment); * *P *= 0.046 versus 6-OHDA-treated group; *** *P *< 0.0001 versus 6-OHDA-treated group. All experiments were repeated 3 times.

All the tyrosine hydroxylase (TH)-positive neurons in zebrafish diencephalons are dopaminergic neurons [21]. Anti-TH immunostaining was used to compare the viability of dopaminergic neurons in zebrafish receiving different drug treatments (Figure 2). 2 dpf zebrafish were exposed to 250 μM 6-OHDA for 24 hours, dopaminergic neurons in the ventral diencephalic clusters (indicated by white bracket) were significantly reduced when compared to the control. Co-treatment with 6-OHDA and EBE for 24 hours recovered 6-OHDA-induced dopaminergic neuron loss in a dose-dependent manner (*P *= 0.046 at 25 μg/ml, *** *P *< 0.0001 at 25 and 50 μg/ml compared with 6-OHDA treatment alone) (Figure 3). Nomifensin (DAT inhibitor) and L-NAME were used as positive controls, both significantly reversed the loss of dopaminergic neurons caused by 6-OHDA (*P *< 0.0001).

### Inhibition of 6-OHDA-induced decrease in total distance of movement in zebrafish

**Figure 4:**
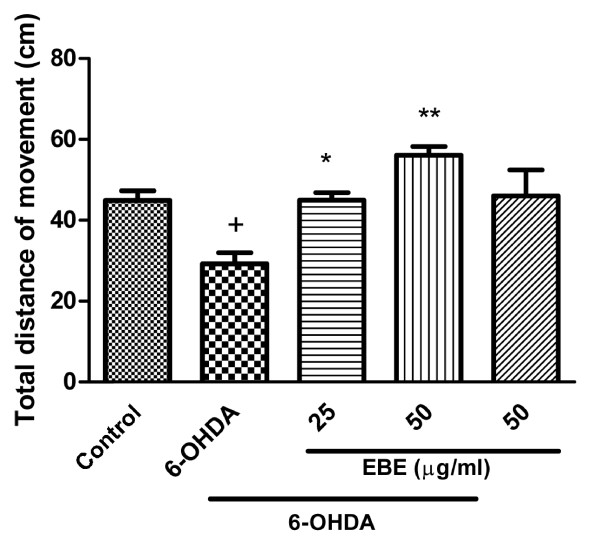
**EBE inhibited 6-OHDA-induced decrease of total distance of movement in zebrafish**. Quantification of the swimming parameter of zebrafish: 4 dpf wild type zebrafish larvae were treated with 6-OHDA (250 μM) and EBE (25 and 50 μg/mL) for three days. The locomotor activity of zebrafish was assessed at seven dpf. All results were expressed as total distance of movement traveled by the larvae, ^+ ^*P *= 0.032 versus control group (without 6-OHDA treatment); * *P *= 0.041, ** *P *= 0.007 versus 6-OHDA-treated group. All experiments were repeated 3 times.

The total distance of movement by 6-OHDA-lesioned fish was significantly decreased when compared with the control group. EBE inhibited 6-OHDA-induced reduction of total distance of movement in zebrafish in a dose dependent manner (Figure 4).

### Dose-dependent reduction of 6-OHDA-induced cell death in PC12 cells

**Figure 5:**
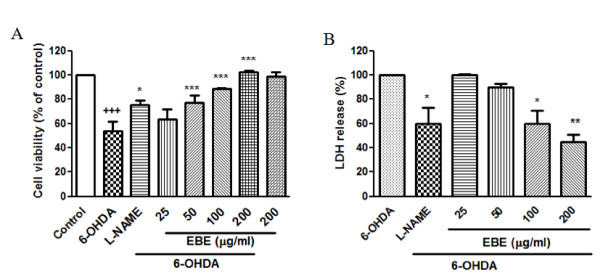
**EBE dose-dependently reduced PC12 cell death induced by 6-OHDA**. PC12 cells were pretreated with or without EBE for 12 hours, pretreatment with 250 μM L-NAME for 12 hours served as positive control. The cells were exposed to 1 mM 6-OHDA for another 12 hours after pretreatment. (A) Cell viability was measured by MTT assay and results were expressed as percentage of control group (without 6-OHDA treatment). (B) Cell viability was measured as the percentage of released LDH. ^+++ ^*P *< 0.0001 versus control group (without 6-OHDA treatment); * *P *= 0.028 versus 6-OHDA group; *** *P *< 0.0001 versus 6-OHDA group. Each experiment was repeated 3 times.

The cell viability of PC12 cells exposed to 1 mM 6-OHDA for 12 hours was significantly decreased (46.2% ± 9.2%; *P *< 0.0001) compared with the control group (Figure 5A). Pretreatment with EBE of various concentrations (25, 50, 100 and 200 μg/ml) for 12 hours protected PC 12 cells against 6-OHDA-induced cellular damage in a dose-dependent manner. Compared with the control, the survival rates of the EBE treatment groups (25, 50, 100, 200 μg/ml) were 63.4% ± 7.3%, 77.1% ± 4.5%, 88.8% and 102.3% respectively. Toxicity was not observed when the cells were treated with 200 μg/mL alone. LDH is released from the cells following membrane damage, as a sign of cell death. Evaluation of LDH release revealed a significant increase after 6-OHDA exposure in PC12 cells while pretreatment with EBE suppressed the 6-OHDA-induced LDH release in a dose dependent manner (Figure 5B). L-NAME is an inhibitor of NOS and served as a positive control. Significant protective effects were found in the positive control groups with both MTT and LDH assays (Figures 5A and 5B).

### Suppression of 6-OHDA-induced apoptosis in PC12 cells

**Figure 6:**
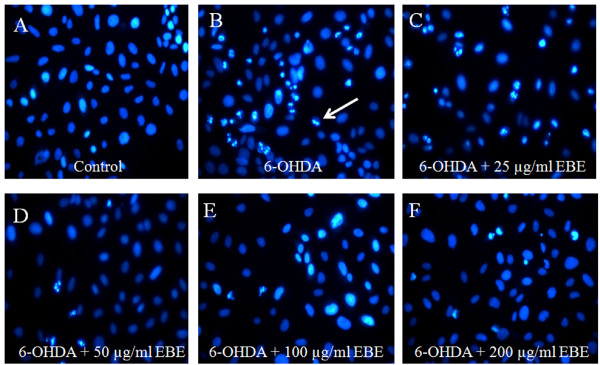
**EBE reduced apoptosis induced by 6-OHDA in PC12 cells**. Cells were stained with DNA-binding fluorescent dye Hoechst 33342. (A) Control: untreated group; (B) 6-OHDA-treated group (1 mM, 8 hours): chromatin condensation and DNA fragmentation were indicated by the white arrows; (C-F) EBE-pretreated groups (25, 50, 100 and 200 μg/mL respectively, 12 hours), followed by 6-OHDA exposure (1 mM, 8 hours): less apoptotic bodies were identified, colony reduction and cell shrinkage induced by 6-OHDA were also reversed.

Apoptosis is morphologically characterized by cell shrinkage, chromatin condensation and nuclear fragmentation. To identify whether EBE reverses 6-OHDA-induced PC12 cell apoptosis, we used DNA staining with Hoechst 33342 to evaluate nuclear condensation. Normal untreated cells appeared circle or elliptical where no condensation of the nucleus was observable (Figure 6A). In contrast, bright condensed dots known as apoptotic bodies (indicated by arrows in Figure 6B) were clearly identified after exposure to 1 mM 6-OHDA for eight hours. Apoptotic bodies are generated when chromatin fragments are packaged in apoptotic cells, and are commonly accepted as a marker of apoptosis. Reductions in colony density and cell size were also observable when treated with 6-OHDA. These changes in nuclear characteristics of apoptosis were inhibited when the cells were pretreated with EBE of different concentrations (25, 50, 100, 200 μg/ml) (Figures 6C-F).

### Inhibition of 6-OHDA-induced NO over-production and down-regulated iNOS over-expression

**Figure 7:**
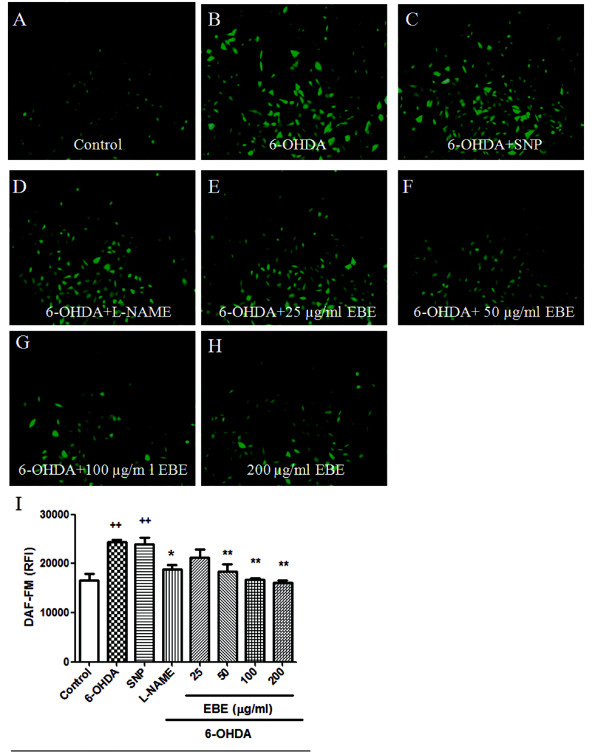
**EBE inhibited 6-OHDA-induced nitric oxide (NO) over-production in PC12 cells**. PC12 cells were pretreated with or without 250 μM L-NAME, 100 μM SNP and 25, 50, 100 and 200 μg/mL EBE for 12 hours, then exposed to1 mM 6-OHDA for another hour. (A-H) Intracellular NO was identified using fluorescent indicator, DAF-FM diacetate; (I) The NO fluorescent intensity was quantified by a multi-label counter. ^++ ^*P *= 0.008 versus control group (without 6-OHDA treatment); * *P *= 0.044 versus OHDA group; ** *P *= 0.005 versus OHDA group. All experiments were repeated 3 times.

**Figure 8:**
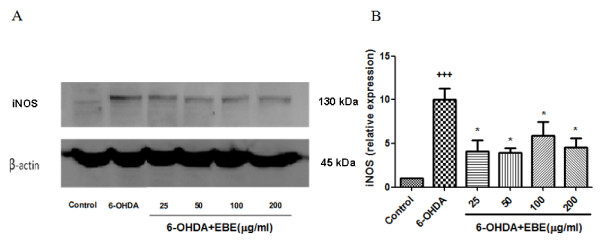
**EBE down-regulated iNOS over-expression in PC12 cells stimulated by 6-OHDA**. Cells were incubated with EBE (25, 50, 100 and 200 μg/ml) for 12 hours prior to a 6-hour 6-OHDA stimulation. (A) Western blot analysis showing 6-OHDA-induced iNOS over-expression was inhibited by pretreatment of different concentrations of EBE. (B) Densitometric analysis of iNOS expression with measurements of each blot expressed relative to that of the control. Three times independent experiments showed the same tendency of the iNOS expression.

Figure 7 shows that 6-OHDA exposure led to a roughly 1.5-fold increase in NO production compared with the control group; similar elevation was also observed when SNP (sodium nitroprusside dehydrate, NO generator) was added. This increase in NO level was reduced in a concentration-dependent manner by pretreatment with EBE in a 25-200 μg/mL range for 12 hours (Figure 7E-H). Pretreatment with 250 μM L-NAME for 12 hours also significantly (*P *= 0.044) reduced this 6-OHDA-induced NO over-production. Pretreatment with higher concentrations (50, 100, 200 μg/ml) of EBE suppressed the elevated NO production more efficiently than L-NAME (Figure 7D). Furthermore, DAF-FM diacetate staining was photographed with a fluorescent microscope (Figure 7A-H). The expression of iNOS was up-regulated by 6-OHDA exposure in Western blot analysis and such up-regulation was prevented by pretreatment with EBE (Figure 8A).

### Further studies

Many flavonoids, such as those derived from *Vitis vinifera *(grape), *Camellia sinensis *(tea), *Theobroma cacao *(cocoa) and *Vaccinium *spp. (blueberry), exert their neuroprotective actions *via *(1) modulating intracellular signaling cascades controlling neuronal survival, death and differentiation, (2) affecting gene expression and (3) interacting with mitochondria [22]. The neuroprotective effects of volatile oils have also been reported; for example, curcuma oil modulates the NO system response to cerebral ischemia/reperfusion injury in rats [23]. As EBE is the ethanol extract of *Ericaulon buergerianum *which is rich in flavonoids and volatile oils, flavonoids and volatile oils may be the active ingredients in EBE. Further studies are warranted to confirm this.

## Conclusion

EBE exhibited significant neuroprotective activities in zebrafish, including recovery of dopaminergic neuron loss caused by 6-OHDA in a dose-dependent manner *in vivo*, inhibition of 6-OHDA-induced decrease of total distance in movement in zebrafish. The iNOS-NO pathway may be involved.

## Abbreviations

EBE: *Ericaulon buergerianum *ethanol extract; F-12K: Kaighn's modification of Ham's F12 medium; MTT: 3-[4, 5-dimethyl- thiazol-2-yl]-2, 5-diphenyl tetrazolium bromide; FBS: Fetal bovine serum; DMSO: dimethyl sulfoxide; PBS: phosphate-buffered saline; 6-OHDA: 6-hydroxydopamine; NO: Nitric oxide; L-NAME: L-nitroarginine methylester; SNP: sodium nitroprusside dehydrate; CNS: central nervous system; PD: Parkinson's disease; SNpc: substantia nigra pars compacta; dpf: day post fertilization.

## Competing interests

The authors declare that they have no competing interests.

## Authors' contributions

MWW and ZJZ performed the experiments. MWW wrote the manuscript. ZXL reviewed the literature and study design. LCVC revised the manuscript. SMYL supervised the study. All authors read and approved the final version of the manuscript.

## References

[B1] Soto-Otero R Mendez-Alvarez E Hermida-Ameijeiras A Munoz-Patino AM Labandeira-Garcia JL Autoxidation and neurotoxicity of 6-hydroxydopamine in the presence of some antioxidants: potential implication in relation to the pathogenesis of Parkinson's disease J Neurochem 2000 74 1605 1612 10737618 10.1046/j.1471-4159.2000.0741605.x

[B2] Elkon H Melamed E Offen D Oxidative stress, induced by 6-hydroxydopamine, reduces proteasome activities in PC12 cells: implications for the pathogenesis of Parkinson's disease J Mol Neurosci 2004 24 387 400 10.1385/JMN:24:3:387 15655261

[B3] Gomes MZ Raisman-Vozari R Del Bel EA A nitric oxide synthase inhibitor decreases 6-hydroxydopamine effects on tyrosine hydroxylase and neuronal nitric oxide synthase in the rat nigrostriatal pathway Brain Res 2008 1203 160 169 18313645 10.1016/j.brainres.2008.01.088

[B4] Riobo NA Schopfer FJ Boveris AD Cadenas E Poderoso JJ The reaction of nitric oxide with 6-hydroxydopamine: implications for Parkinson's disease Free Radic Biol Med 2002 32 115 121 10.1016/S0891-5849(01)00758-4 11796199

[B5] Noh EJ Ahn KS Shin EM Jung SH Kim YS Inhibition of lipopolysaccharide-induced iNOS and COX-2 expression by dehydroevodiamine through suppression of NF-kappaB activation in RAW 264.7 macrophages Life Sci 2006 79 695 701 10.1016/j.lfs.2006.02.020 16554073

[B6] Parathath SR Gravanis I Tsirka SE Nitric oxide synthase isoforms undertake unique roles during excitotoxicity Stroke 2007 38 1938 1945 10.1161/STROKEAHA.106.478826 17446423

[B7] Kindler DD Thiffault C Solenski NJ Dennis J Kostecki V Jenkins R Keeney PM Bennett JP Jr Neurotoxic nitric oxide rapidly depolarizes and permeabilizes mitochondria by dynamically opening the mitochondrial transition pore Mol Cell Neurosci 2003 23 559 573 10.1016/S1044-7431(03)00074-5 12932437

[B8] Boje KM Nitric oxide neurotoxicity in neurodegenerative diseases Front Biosci 2004 9 763 776 10.2741/1268 14766406

[B9] Ho JC Chen CM Flavonoids from the aquatic plant Eriocaulon buergerianum Phytochemistry 2002 61 405 408 10.1016/S0031-9422(02)00218-2 12377234

[B10] Chinese Pharmacopoeia Commission Pharmacopoeia of the People's Republic of China 2005 1 Beijing: Chemical Industry Press21032922

[B11] Fang JJ Ye G Chen WL Zhao WM Antibacterial phenolic components from Eriocaulon buergerianum Phytochemistry 2008 69 1279 1286 10.1016/j.phytochem.2007.11.014 18191163

[B12] Yang WC Liu H Chen QL Gujingcaoke yaoxue yanjiu gaikuang Acta Chin Medicine and Pharmacol 2009 37 92 93

[B13] Sun YX Zhang DF Guqingtang jianjie HeNan Tradit Chin Med 2001 21 31

[B14] Parng C Roy NM Ton C Lin Y McGrath P Neurotoxicity assessment using zebrafish J Pharmacol Toxicol Methods 2007 55 103 112 10.1016/j.vascn.2006.04.004 16769228

[B15] McKinley ET Baranowski TC Blavo DO Cato C Doan TN Rubinstein AL Neuroprotection of MPTP-induced toxicity in zebrafish dopaminergic neurons Brain Res Mol Brain Res 2005 141 128 137 16209898 10.1016/j.molbrainres.2005.08.014

[B16] Rink E Wullimann MF Connections of the ventral telencephalon and tyrosine hydroxylase distribution in the zebrafish brain (Danio rerio) lead to identification of an ascending dopaminergic system in a teleost Brain Res Bull 2002 57 385 387 10.1016/S0361-9230(01)00696-7 11922994

[B17] Anichtchik OV Kaslin J Peitsaro N Scheinin M Panula P Neurochemical and behavioural changes in zebrafish Danio rerio after systemic administration of 6-hydroxydopamine and 1-methyl-4-phenyl-1,2,3,6-tetrahydropyridine J Neurochem 2004 88 443 453 14690532 10.1111/j.1471-4159.2004.02190.x

[B18] Panula P Sallinen V Sundvik M Kolehmainen J Torkko V Tiittula A Moshnyakov M Podlasz P Modulatory neurotransmitter systems and behavior: towards zebrafish models of neurodegenerative diseases Zebrafish 2006 3 235 247 10.1089/zeb.2006.3.235 18248264

[B19] Institute of Materia Medica CAoMS Zhong Yao Zhi 1995 Beijing: People's Medical Publishing House

[B20] Westerfield M The Zebrafish Book: A Guide for the Laboratory Use of Zebrafish (Danio rerio) 2007 5 Eugene: University of Oregon Press

[B21] Schweitzer J Driever W Development of the dopamine systems in zebrafish Adv Exp Med and Biol 2009 651 1 14 10.1007/978-1-4419-0322-8_1 19731546

[B22] Spencer JP Flavonoids: modulators of brain function? Br J Nutr 2008 99E Suppl 1 ES60 77 10.1017/S0007114508965776 18503736

[B23] Dohare P Varma S Ray M Curcuma oil modulates the nitric oxide system response to cerebral ischemia/reperfusion injury Nitric Oxide 2008 19 1 11 18485279 10.1016/j.niox.2008.04.020

